# Improvement of Bioactive Components and Technological Quality of Gluten-Free Pasta with Utilization of Different Carrot Powders, Guar Gum and Pregelatinization Application

**DOI:** 10.3390/foods13244101

**Published:** 2024-12-18

**Authors:** Hilal Arslan Bayrakcı, Nermin Bilgiçli

**Affiliations:** 1 Karapınar Aydoğanlar Vocational School, Selçuk University, 42400 Konya, Turkey; 2 Department of Food Engineering, Engineering Faculty, Necmettin Erbakan University, Köyceğiz Campus, 42050 Konya, Turkey; nerminbil2003@hotmail.com

**Keywords:** celiac, gluten-free pasta, carrot, pregelatinization, guar gum

## Abstract

In this study, carrot (orange and black) powder substitution (0–15%) and different dough applications (guar gum (GG) addition, pregelatinization (PG) and a PG + GG combination) were researched in gluten-free pasta preparation to improve the bioactive components and technological properties. Some quality attributes and bioactive components of the pasta were determined. Black carrot powder substitution into the pasta revealed rich functional properties with higher total dietary fiber (TDF), Ca, K, Mg, P and total phenolic content (TPC) than orange carrot powder. An increased carrot powder addition ratio in the gluten-free pasta formulation resulted in enrichment in ash, mineral, β-carotene, total anthocyanin, TDF, antioxidant activity and TPC. The amounts of β-carotene and anthocyanin in the pasta samples rose to 4.42 mg/100 g and 26.08 mg CGE/100 g with the addition of 15% orange and black carrot powders, respectively. Increasing cooking loss due to high utilization ratios of carrot powder was eliminated by PG and PG + GG applications, and technologic quality was improved, especially with the PG + GG combination.

## 1. Introduction

Pasta is a cereal product widely consumed by people of all ages around the world. In recent years, gluten-free pasta products have been consumed not only by an increasing number of people with celiac disease but also by people wishing to eliminate gluten-based products from their diets for health reasons. The main problems with gluten-free products are poor nutritional and functional properties due to their starch-based nature and poor product quality due to the lack of gluten. Various sources of protein, dietary fiber, minerals, vitamins and phytochemicals can be added to enhance the nutritional and functional properties of this product. To solve the technological quality problems caused by gluten deficiency, modified starches, heat-treated flours and hydrocolloids are preferred in product formulation. This study incorporates chickpea flour and orange/black carrot powders into pasta to enhance its nutritional and functional properties while utilizing GG and PG to improve the technological quality of gluten-free pasta.

Carrot (*Daucus carota* L.), one of the most important root vegetables of the Apiaceae family, is cultivated worldwide [1]. Because of its richness in carotenoids, anthocyanins, dietary fiber, vitamins and other nutrients, storage root is widely used [2,3]. Carrots can be divided into two groups according to their color pigments: the anthocyanin group (*Daucus carota* subsp. *Sativus* var. *Atrorubans*) and the carotene group (*Daucus carota* ssp. *Sativus*) [4]. About 60% of ripe orange carrots’ carotenoid content is β-carotene, 20% is α-carotene, with the rest comprising lycopene, γ-carotene, ζ-carotene and/or β-zeacarotene [5]. Anthocyanins, an important subgroup of flavonoids, are water-soluble plant pigments responsible for the blue, purple and red colors of many plant tissues [6]. Anthocyanins are known for their ability to scavenge free radicals that can cause oxidative stress in human cells [7]. Black carrot is a root vegetable with a very high content of anthocyanins. The amount of anthocyanin in the roots can vary from trace amounts in pink varieties to 1750 mg/kg in black carrots [6].

Guar gum (GG) is a plant polysaccharide composed of galactose and mannose found in the endosperm of guar seeds [8]. The structure of GG consists of a linear chain of (1 → 4)-β-D-mannopyranose units and a side chain of α-D-galactosyl pyranose units linked by (1 → 6) bonds [9]. GG is water soluble and hydrophilic and can be used as a thickener and stabilizer in the food, petroleum, paper and textile industries [10,11,12]. PG is a common type of physical modification of starch [13]. Physically modified starch swells rapidly in cold water and has improved viscosity and desirable food textural properties [14]. PG is widely used for dough and noodles [15].

This is the first study on the effect of using different carrot powders with different dough treatments in gluten-free pasta production on the bioactive components and technological quality of pasta. The objectives of this study were (1) to formulate new functional food with the utilization of different carrot powders in gluten-free pasta formulation; (2) to enhance the technological characteristics of gluten-free pasta using GG, PG and PG + GG applications; and (3) to evaluate the physical, chemical and bioactive components of gluten-free pasta prepared by partial substitution of orange and black carrot powders and different dough applications.

## 2. Materials and Methods

### 2.1. Materials

Corn (Bağdat Baharat, Ankara, Turkey), rice (Kenton, İstanbul, Turkey) and chickpea (Doğalsan, Ankara, Turkey) flours were bought from local markets in Konya, Turkey. Orange and black carrots were sourced from the town of Kaşınhanı in Konya, Turkey. Guar gum was procured from Vatan Chemistry and Machinery Industry. Tic Ltd., Şti. (Istanbul, Turkey).

#### 2.1.1. Carrot Powder Production

The carrots were cleaned with tap water, and their skins were shaved. The carrots were sliced thin and blanched in boiling water at 90 °C for 3 min. The carrots treated with heat were dried at 60 °C for 10 h [16]. The dried carrots were ground with a laboratory mill (Arçelik-K3104) and stored in air-light-proof containers under refrigerator conditions.

#### 2.1.2. PG Application

The PG process was applied to 25% of the flour blend in all gluten-free pasta formulations. For PG, two times of boiling water of the flour blend was added to the flour blend and kept in the boiling water bath for 5 min. These samples were used in gluten-free pasta production after being kept at room temperature for 2 h [17].

### 2.2. Methods

#### 2.2.1. Gluten-Free Pasta Production

Gluten-free pasta samples were prepared by modifying the method of Susanna and Prabhasankar [18]. The gluten-free pasta formulations are presented in Supplementary Table S1. Gluten-free pasta without carrot powder was produced with 400 g corn flour, 400 g rice flour and 200 g chickpea flour. For the preparation of gluten-free pasta by PG application, 25% of the flour blend used in the preparation of the gluten-free pasta was pregelatinized. For the production of pasta with gum, 3% GG was used. In the samples where PG + GG were applied together, GG was added into the flour blend after PG application. The amount of water used for pasta preparation was determined as a result of preliminary trials. Orange carrot powder or black carrot powder was used at 0, 5, 10 and 15% ratios as replacing with corn flour:rice flour blend. On the other hand, chickpea flour was used at a fixed ratio (20%) in all formulations. The gluten-free pasta experiments were carried out in two replications (4 × 2 × 3) × 2, according to carrot powder type (CPT) (×2), carrot powder addition ratio (CPR) (×4) and applications method (×3) trial design. A short cut penne die was preferred in pasta shaping. The pasta ingredients were kneaded on the kneading pallet of the pasta production machine (La Monferrina, Dolly, Italy) for about 20 min and then extruded. A short cut penne die was preferred for pasta forming. The fresh pasta samples were dried in a dryer (La Monferrina, EC25, Italy) at a maximum temperature of 58 °C and stored for analysis in an appropriate temperature and humidity environment.

#### 2.2.2. Chemical and Bioactive Component Analysis

Before chemical analysis, the dry pasta was ground and passed through a 500 µ sieve. AACC standard methods were used for the determination of the ash (AACC 08-01-01), protein (46-12.01) and fat (30-25.01) contents of the gluten-free pasta samples [19]. TDF analysis of the samples was conducted according to the method of AOAC 991.43 [20]. Grinded dry samples were subjected to sequential enzymatic digestion with α-amylase, protease and amyloglycosidase to remove starch and protein. The enzyme digestion product was treated with alcohol to precipitate soluble dietary fiber before filtering, and the TDF residue was washed with alcohol and acetone and then dried and weighed.

The samples’ TPC was measured colorimetrically using the Folin–Ciocalteau method. An acidified methanol solution (20 mL, HCl:methanol:water, 1:80:10, *v*/*v*) was added to 4 g of sample in the test tube, and the tubes were shaken in a shaking water bath (24 ± 1 °C) and then centrifuged (3000× *g* rpm, 10 min). The obtained supernatants were used in the TPC analysis [21,22]. After centrifugation, 0.1 mL of the upper liquid was moved into a test, and Folin–Ciocaltaeu reagent (0.5 mL, 10% *v*/*v*, in water) and sodium carbonate solution (1.5 mL, 20% *w*/*v*, in water) were added and incubated at room temperature for 2 h. The absorbance value of the final solution, which was prepared and incubated at the end of the period, was read with the help of a UV-spectrophotometer (Hitachi-U1800, Hitachinaka, Ibaraki 312-8504, Japan) at a wavelength of 760 nm. The TPC contents of the samples were calculated as gallic acid equivalents [23,24]. The results were expressed as mg of gallic acid equivalents per 100 g.

For determining the antioxidant activities of the samples, the DPPH method was used [22,25]. The samples were extracted as in the TPC assay, and the samples were treated with DPPH (2,2-diphenyl-1-picrylhydrazil). The absorbance value of the obtained solution was measured with a UV-spectrophotometer (Hitachi-U1800, Hitachinaka, Ibaraki 312-8504, Japan) at a wavelength of 517 nm. The results were evaluated according to the following formula:

%Inhibition: [(Ab_control_ − Ab_sample_)/Ab_control_] × 100

Ab_control_: Absorbance value of the control sample

Ab_sample_: Absorbance value of the pasta sample

For the determination of calcium (Ca), iron (Fe), potassium (K), magnesium (Mg), phosphorus (P) and zinc (Zn) in the samples, a 1 g dry sample was utilized. This sample was dissolved using the wet digestion technique in a microwave combustion system (Mars 5, CEM Corporation, Matthews, NC, USA) with a combination of 10 mL sulfuric acid and nitric acid. The levels of the mineral compounds in the obtained filtrates were determined using an inductively coupled plasma atomic emission spectrophotometer (ICP-AES) device (Vista Series, Varian International AG, Zug, Switzerland).

The β-carotene concentration in the samples was assessed based on Prakash et al. [26], with some modification of the method. Accordingly, dry samples were treated with hexane:acetone:ethanol mixed organic solvents, and the process was continued until the residue became colorless. The obtained orange-colored liquid was filtered into a separatory funnel, and petroleum ether and sodium sulfate were added to the liquid. After shaking for 1 min, two separate phases were formed in the funnel. The absorbance of the supernatant was read in a UV-spectrophotometer (Hitachi-U1800, Hitachinaka, Ibaraki 312-8504, Japan) at a wavelength of 452 nm. Petroleum ether containing 3% acetone was used as the blank. The β-carotene standard curve was obtained within the range of 0–5 µg β-carotene per ml, and the calculation was made by plotting the curves of the absorbance values against the concentrations. The results are given as mg/100 g of β-carotene.

The total anthocyanin content in the samples was analyzed using the method described by Ficco et al. [27]. A mixture of methanol (8 mL) acidified with 1 N HCL (85:15, methanol:HCl, *v*/*v*) was added to 0.5 g of sample in 50 mL centrifuge tubes and kept in an ultrasonic water bath for 18 min at room temperature (Bandelin Sonorex, Berlin, Germany). After the samples were centrifuged at 9000× *g* for 15 min, the supernatants were taken into 15 mL centrifuge tubes. The precipitate was again treated with 4 mL of acidified methanol, and the procedure was repeated. The obtained supernatants were incubated for 48 h in the dark at −20 °C to facilitate macromolecule precipitation. The samples from the incubation were centrifuged again and filtered with 0.45 µm regenerated cellulose syringe filters. The total anthocyanin content was evaluated using a calorimetric method with different pH solutions. The extracted supernatants (750 µL) were added to two separate tubes and diluted with potassium chloride buffer solution (0.03 M) for pH 1 and sodium acetate buffer solution (0.4 M) for pH 4.5. The samples, which were incubated for 30 min at room temperature in dark conditions, were filtered again, and the absorbance values were read against the blank sample with distilled water at 520 and 760 nm wavelengths. Total anthocyanin content was calculated as cyanidin-3-glucoside equivalents (mg CGE/100 g).

#### 2.2.3. Color Measurement

The color of the gluten-free pasta samples was measured using a Hunter Lab Chroma Meter (Minolta CR-400, Osaka, Japan). Color values were evaluated according to the CIE L*a*b* color coordinate system of the International Commission on Illumination. Chroma (C*, color intensity) value was calculated with the formula [a^2^ + b^2^]^1/2^ [28], and Hue angle (a* > 0 and b* > 0, if arctan [b*/a*]; a* > 0 and b* < 0, arctan [b*/a*] + 360°) values were calculated.

#### 2.2.4. Cooking Characteristics and Firmness

Weight and volume increase values of the gluten-free pasta samples were determined according to [29,30]. For this purpose, firstly, the cooking times of the gluten-free pasta samples were determined with preliminary experiments. Then, 20 g of a gluten-free pasta sample was cooked in 250 mL of boiling distilled water for the specified cooking time. The differences in weight between uncooked and cooked gluten-free pasta specimens were represented as a percentage. For the volume increase test, the cooked and drained gluten-free pasta specimens were placed in graduated cylinders filled with distilled water. The amount of displaced liquid was measured to determine the values for volume increase. Cooking loss was evaluated by filtering 20 g of gluten-free pasta that had been boiled in 250 mL of hot distilled water The liquid collected was subsequently dried in an oven at 135 °C.

The firmness of the pasta samples was evaluated by means of the A/LKB-F blade hardware in the TAXTPlus texture analysis device (Stable Microsystems, Surrey, UK) according to the method given by Yeyinli [31].

#### 2.2.5. Statistical Analysis

The statistical data analysis was conducted using the Minitab 16 program. The obtained data were subjected to analysis of variance. Duncan’s multiple comparison test was used to compare the means of the main variance sources with the MstatC statistical program.

## 3. Results and Discussion

### 3.1. Chemical Properties and Bioactive Components of Gluten-Free Pasta Samples

#### 3.1.1. Ash, Protein and Fat Contents

The chemical analysis results of the gluten-free pasta samples are shown in Table 1 and Table S5. The results were discussed separately in terms of variance sources (carrot powder type, carrot powder ratio and application method) according to Duncan’s multiple comparison test results (Table 1). When comparing the results based on the carrot powder type, it was found that the ash, protein and fat contents of the gluten-free pasta did not exhibit significant changes. According to the carrot powder ratio variance source, an increasing carrot powder ratio also increased the mean ash content of the gluten-free pasta from 1.03% up to 1.78%. On the other hand, the protein content of the gluten-free pasta decreased from 12.39% to 11.71% with 15% carrot powder addition. The fat content of the gluten-free pasta did not change significantly with the carrot powder addition ratio.

As seen in Supplementary Table S2, the ash, protein and fat contents of the orange and black carrot powders used as raw materials in gluten-free pasta preparation were found to be 5.71%, 7.93% and 2.16% (orange); and 5.88%, 8.49% and 2.55% (black), respectively. In the gluten-free pasta formulation, carrot powders were used by replacing the rice flour:corn flour blend at different ratios. The amounts of ash, protein and fat in corn flour and rice flour were found to be 0.65%, 9.0% and 1.71% (corn); and 0.42%, 10.79% and 0.59% (rice), respectively (Supplementary Table S2). Especially, the ash content of both carrot powders, which was higher than corn and rice flour, was also effective in increasing the ash content of the gluten-free pasta samples. Porto Dalla Costa et al. [32] determined the protein and fat contents of control pasta and pasta samples containing 20% carrot powder as 9.60% and 0.21% (control); and 11.80% and 2.71%, respectively. In a study examining the chemical properties of tortillas produced with the addition of carrot powder (10%), it was reported that carrot powder tortillas contained 60–70% more ash content than the control sample [33]. In another study in which carrot juice, puree and pomace were used in pasta making, it was found that the amount of protein in the carrot pasta was lower than the control pasta, and the amount of ash in the carrot pasta was higher than the control pasta [34].

When the results were evaluated according to the application method variance source, the protein and fat contents of the gluten-free pasta were not affected by the application method. However, there was a statistical difference in ash content, with the lowest amount of ash observed in the gluten-free pasta produced using the PG application. The total ash content of GG was determined to be between 3.05% and 3.52% by Kays et al. [35]. The higher ash content of GG-substituted samples may originate from the high ash content of GG.

#### 3.1.2. TDF Contents

Fiber from vegetables has a role in trapping harmful toxins in the intestinal tract, lowering serum LDL cholesterol without affecting HDL cholesterol, reducing fat absorption, lowering the glycemic index, reducing the risk of cardiovascular disease and acting as a prebiotic for beneficial microbiota in the intestinal tract [36,37].

The TDF content of the pasta prepared with black carrot powder was higher than that of the pasta containing orange carrot powder (Table 1). The higher TDF content of black carrot powder (42.92%) compared to orange carrot powder (29.27) may have been effective in obtaining this result (Supplementary Table S2).

When the results were evaluated according to the carrot powder ratio variance source, increasing the carrot powder ratio significantly (*p* < 0.05) increased the TDF content of the gluten-free pasta. The TDF content of the gluten-free pasta containing 15% carrot powder was 57% higher than that of the gluten-free pasta without carrot powder. Considering that gluten-free products are generally starch-based with low fiber content, this TDF increase with the use of carrot powder was found to be significant in terms of healthy nutrition for celiac patients. On the other hand, the TDF contents of the corn flour and rice flour used in the control gluten-free sample in this study were found to be 4.49 and 2.78%, respectively (Supplementary Table S2). It was reported in a study that the cereal product produced with the addition of 10% carrot powder contained 35% more TDF than the control sample [33]. Wang [34] determined the amount of TDF to be 11.46 g/100 g in the pasta sample to which 10% carrot pomace was added, while this ratio was 3.60 g/100 g in the control pasta.

The TDF contents of the gluten-free pasta prepared by GG, PG and PG + GG applications were found to be 10.79, 8.66 and 10.99%, respectively. Both of the GG-added gluten-free pasta samples were found to have a higher TDF content than the PG-applied sample. The high fiber content of GG may have been effective in achieving this result. It has been reported that the TDF amount of GG constitutes 52–58% of the seed dry weight [35].

#### 3.1.3. Antioxidant Activity and TPC

The gluten-free pasta samples containing black carrot powder exhibited higher antioxidant activity and TPC values than the pasta samples prepared using orange carrot powder (Table 1 and Table S5). The antioxidant activity and TPC contents of the orange and black carrot powders were found to be 75.16% and 263.50 mg GAE/100 g; and 87.52% and 596.10 mg GAE/100 g, respectively (Supplementary Table S2). The high antioxidant properties of anthocyanin pigments that give black carrots their color may have caused this situation. Black carrot is a good source of anthocyanins (1750 mg/kg), mostly composed of acylated anthocyanins, and anthocyanins are also a powerful antioxidant [38,39]. Moreover, the TPC of black carrot powder was found to be 2.3 times higher than that of orange carrot powder. Both the higher antioxidant activity and TPC content of black carrot powder compared to those of orange carrot powder were also reflected in the pasta.

Regarding the carrot powder ratio, the antioxidant activity values of the gluten-free pasta samples with 0, 5, 10 and 15% carrot powder were determined to be 5.72, 33.42, 41.30 and 51.09%, respectively. The antioxidant activity values of the pasta samples increased significantly as the carrot powder ratio increased. The antioxidant activity value of the pasta sample with 15% carrot powder addition ratio was approximately nine times higher than that of the pasta without carrot powder. The highest carrot powder ratio (15%) also increased the TPC of the gluten-free pasta samples from 10.37 to 67.97 mg GAE/100 g, and there was a 6-fold increase. Carrot pigments, such as carotenoids and flavonoids, have rich antioxidant properties in addition to color. Kamiloglu et al. [40] determined significant increases in the TPC and antioxidant activity amounts of cake samples containing black carrot pulp powder (0–15%). Olawuyi and Lee [41] investigated the TPC and antioxidant activity of rice cakes substituted with 5, 10 and 15% carrot powder. The antioxidant activity and TPC were found to be 9.71% and 12.27 mg GAE/100 g in the control sample using complete rice flour, and these values reached to 49.31% and 34.21 mg GAE/100 g in the sample with 15% carrot powder. Pekmez and Bay Yılmaz [42] compared the TPC content of breads obtained by using black carrot flour (1%, 2.5, 5 and 7.5%) with the control samples and observed that it was approximately eight times higher for the crust and nine times higher for the crumb.

Concerning the application method factor, the PG + GG application gave a higher TPC value than PG application alone. There was no statistical change in the antioxidant activity values according to application method.

#### 3.1.4. Mineral Contents

The gluten-free pasta samples with black carrot powder were found to have higher mineral (except Fe and Zn) contents than the samples containing orange carrot powder (Table 2 and Table S6). The Ca, Fe, K, Mg, P and Zn contents for the orange and black carrot powders used as raw materials were 403.48 mg/100 g, 1.30 mg/100 g, 1630.90 mg/100 g, 224.23 mg/100 g, 293.19 mg/100 g, 2.92 mg/100 g and 418.13 mg/100 g; and 1.89 mg/100 g, 2296.90 mg/100 g, 296.00 mg/100 g, 361.48 mg/100 g and 1.42 mg/100 g, respectively (Supplementary Table S3). J.P. Singh et al. [43] reported that black carrot contains two times more K and seven times more Ca than orange carrot.

As the ratio of carrot powder was raised, significant (*p* < 0.05) increases in the amount of all minerals except Fe and Zn were observed. Compared to the control pasta without carrot powder, the Ca, K, Mg and P contents of the pasta prepared with the highest carrot powder ratio (15%) increased by 3.2, 1.9, 1.4 and 1.0 times, respectively. The lower mineral content of corn flour and rice flour compared to that of carrot powders was effective in obtaining these results (Supplementary Table S3). Sule et al. [44] found significant increases in the Ca, Fe, K and Na contents of pasta prepared by replacing 5, 10, 15, 20, 25 and 30% carrot powder with wheat flour.

In general, there were small numerical changes in the mineral substance amounts of the gluten-free samples according to the application method factor. Statistically, lower Ca and K amounts were obtained in the gluten-free pasta samples with PG (alone) compared to the other treatments. As mentioned before, the high ash content of GG may have been effective in obtaining higher mineral amounts compared to the PG application.

#### 3.1.5. β-Carotene and Total Anthocyanin Contents

β-carotene analysis was performed on the gluten-free pasta samples containing orange carrot powder, while total anthocyanin analysis was conducted on the sample prepared with black carrot powder, owing to the applicability of the used analysis methods. The β-carotene amount of the gluten-free pasta samples prepared using orange carrot powder varied between 2.15 and 4.42 mg/100 g (Table 3). As the orange carrot powder ratio increased, in all application methods, an increase was observed in the amount of β-carotene in the gluten-free pasta samples. The β-carotene content of the orange carrot powder was found to be 40.35 mg/100 g (Bayrakcı Arslan, 2020) [45]. Simões et al. [46] determined the amount of β-carotene in dried orange carrots as 28.3 mg/100 g. Although there are some losses in the amount of β-carotene during drying, orange carrots are an important source of β-carotene. Both in our study and in the literature, it is seen that carrot powders also contain very high levels of β-carotene. The amount of β-carotene in the pasta samples prepared with the PG and PG + GG applications was found to be lower than the amount of β-carotene in the samples prepared with only the GG application. Gayas et al. [47] used carrot pulp powder (2.5, 5, 7.5 and 10 %) in biscuit production and reported a six times increase in β-carotene content with the addition of 10% carrot pulp powder compared to the control. Santana-Gálvez et al. [33] also determined that the amount of β-carotene of a tortilla enhanced significantly with the substitution of 10% carrot flour.

The total anthocyanin amount of the pasta samples prepared with black carrot powder ranged between 15.46 and 26.08 mg CGE/100 g (Table 3). Similarly to the β-carotene results, as the black carrot powder ratio increased, in all application methods, an increase was observed in the total anthocyanin content of the gluten-free pasta samples. The total anthocyanin amount of the black carrot powder was found to be 259.08 mg CGE/100 g (Bayrakcı Arslan, 2020) [45]. The total anthocyanin amount of black carrot pulp has been reported as 446.10 mgCGE/100 g by Kamiloglu et al. [39]. Although there are some differences in the literature for the total anthocyanin amounts of black carrot powder depending on the drying method, generally, very high total anthocyanin values have been reported. The total anthocyanin amount was found to be lower in the gluten-free pasta samples in which the PG application was used alone or in combination with GG compared to the samples in which only GG was applied. Kamiloglu et al. [40] determined significant increases in the total anthocyanin amounts of cake samples substituted with black carrot pulp powder (5, 10 and 15%). Singh et al. [48] determined the anthocyanin amount of noodles containing 10% black carrot flour as 14.94 mg/100 g.

### 3.2. Color Values of Gluten-Free Pasta Samples

As expected, the pasta prepared using black carrot powder exhibited lower L*, a*, b* and C* values than those of the pasta prepared using orange carrot powder additions (Table 4 and Table S7). As can be observed in Supplementary Table S4, L*, a*, and b* values of orange and black carrot powders are 70.81, 19.20 and 35.75; and 39.28, 12.19 and −4.02, respectively. From these values, it is understood that the color properties of the raw material are reflected in the color of the pasta.

According to the carrot powder addition ratio factor, the L* values of the pasta were reduced with increasing carrot powder ratio, but a* values increased. While the black carrot powder was more effective on reducing the brightness of the pasta samples, the orange carrot powder showed a dominant feature on the increase in the redness (Supplementary Table S4). Compared to the pasta sample that did not use any carrot powder, the yellowness of the pasta samples decreased with the addition of carrot powder, but the Hue value increased. Compared to the control gluten-free pasta, a lower C* value was determined in the samples prepared with 5–10% carrot powder, but when the carrot powder ratio increased to 15%, the highest C* value was reached. Orange carrot powder and black carrot powder contain high amounts of beta carotene (40.35 mg %100 g) and anthocyanin (259.08 mgCGE/100 g), respectively (Bayrakcı Arslan, 2020) [45]. Especially, these color pigments cause color changes in pasta prepared by substitution of carrot powder. In the literature, there are studies reporting that the L* value of pasta samples was reduced, while the a* and b* values rose with the addition of orange carrot powder [32,49,50].

When the results are compared in terms of application methods, the samples produced with only the addition of GG showed higher L* and lower a*, b* and C* values than the gluten-free pasta samples produced with the other applications (Table 4). Bazrafshan et al. [51] examined the color properties of cake prepared using GG and reported that the use of GG resulted in an increase in the brightness of the samples. They explained this situation with the smoothing of the surface of the cake as a consequence of the GG retaining moisture and positively increasing the brightness by affecting the light reflection. In the present study, the use of PG alone or in combination with GG caused an increase in the darkness and redness of the pasta. The heat treatment applied during the PG application may have increased the darkness and redness of the pasta through the Maillard reaction. It has been reported in different studies that the brightness of noodles decreases by PG application in the production of rice and buckwheat noodles [52,53].

### 3.3. Cooking Properties of Gluten-Free Pasta Samples

The cooking quality of the pasta samples is given in Table 5 and Table S8. With the use of black carrot powder in gluten-free pasta formulation, higher weight increase, volume increase, cooking loss and firmness values were obtained compared to orange carrot powder usage in pasta. The higher weight increase and volume increase values of the gluten-free pasta containing black carrot powder may have resulted from the higher TDF content of black carrot powder (42.92%) compared to orange carrot powder (29.27%) (Supplementary Table S2). Moreover, the richer fiber amount of black carrot powder compared to orange carrot powder may have resulted in more weakening of the starch network and increased the amount of cooking loss in the pasta [54].

According to the carrot powder ratio factor, carrot powder usage at a 10–15% ratio gave the highest weight increase, volume increase and cooking loss values among all samples. The firmness value of the pasta samples was found to be higher than the control sample at all carrot powder usage ratios. The increase in weight and volume has been explained in the literature by the increase in the amount of carrot fiber (which has high water affinity) in the pasta formulation, causing more water binding in the pasta [55]. Similarly, the increase in cooking loss in the gluten-free pasta substituted with carrot powder can be explained by the fibers in carrots that may have occurred as the result of uneven water distribution in the pasta due to a tendency toward more hydration [32]. This increase is also related to the weakening of the starch network due to the presence of fiber [56]. Porto Dalla Costa et al. [32] determined the cooking loss value to be 6.43%, 7.88% and 11.71 in pasta samples prepared by adding 0, 10 and 20% carrot powder. Depending on the increasing carrot powder ratio, the amount of cooking loss also increased.

According to the application method variance source, the highest weight increase, volume increase and cooking loss and the lowest firmness were determined in the gluten-free pasta samples prepared with GG addition. From this result, it is understood that GG increased the hydration rate of the pasta, thanks to its water binding ability. The combined use of PG and GG resulted in the lowest weight increase, volume increase and cooking loss values but enhanced pasta firmness. The decrease in cooking loss with the PG application has been explained in the literature; PG causes an increase in the hardness of starch molecules due to the recombination of amylose and/or amylopectin, thus reducing the formation of water-soluble substances [57]. In addition, the PG of flour and starches provides a hard and stable structure when used for product development [58], and PG results in a firmer food texture [59]. Also, in the present study, the PG and PG + GG combination applications gave higher pasta firmness than GG addition. Marti et al. [52] found that gluten-free pasta produced with PG rice flour had less cooking loss and water absorption and higher firmness values compared to pasta obtained from unprocessed rice flour. In a study, gluten-free pasta samples obtained with both PG and gum application were found to have the best cooking properties [17]. In another study examining the effects of PG rice flour and GG application on the textural attributes of pasta, the firmness values of the samples produced with the PG application were higher than the control, and the samples prepared with the PG + GG application gave the highest firmness value [60].

## 4. Conclusions

In the present study, the bioactive components of gluten-free pasta substituted with different carrot powders (0–15%) were revealed. In addition, the effects of GG and PG alone or in combination were researched to enhance the technological quality of gluten-free pasta. The ash, protein, fat and Fe amounts of the pasta samples were not affected by carrot powder type. Higher TDF, antioxidant activity, TPC, Ca, K, Mg and P contents were obtained with black carrot powder substitution compared to orange carrot powder. Only Zn content was found to be lower in the pasta containing black carrot. Black carrot powder substitution resulted in poor pasta quality with respect to pasta color and cooking loss compared to orange carrot powder. An increased carrot powder ratio enhanced the surface darkness, redness, weight increase, volume increase and cooking loss values from the technological quality attributes of the pasta compared to the control. All application methods were significant on the technological properties of the gluten-free pasta. The use of PG alone or in combination with GG was determined as the most effective method for decreasing the cooking loss of the gluten-free pasta.

It is of great importance to ensure nutritional, physical, textural and sensory quality together in gluten-free products such as pasta. As a result of this study, it was revealed that carrot flours are potentially important ingredients in enriching gluten-free pasta with functional ingredients. In addition, it has been observed that successful results can be achieved with the combined use of PG and GG, which are known to contribute to the development of the technological properties of products such as gluten-free pasta. In future studies, the change in functional components during the shelf life of gluten-free pasta containing carrot flour can be researched.

## Figures and Tables

**Table 1:** Duncan’s multiple comparison test results of chemical properties and bioactive components of gluten-free pasta samples.

Variance Source	n	Ash (%)	Protein (%)	Fat (%)	Total Dietary Fiber (%)	Antioxidant Activity (%)	Total Phenolic Content (mgGAE/100 g)
**CPT**							
Orange	24	1.37 ± 0.30 a	12.02 ± 0.27 a	1.96 ± 0.19 a	9.69 ± 1.73 b	28.87 ± 14.46 b	28.70 ± 13.84 b
Black	24	1.38 ± 0.30 a	12.04 ± 0.26 a	1.98 ± 0.15 a	10.60 ± 2.44 a	36.90 ± 20.41 a	49.78 ± 30.82 a
**CPR (%)**							
0	12	1.03 ± 0.01 d	12.39 ± 0.03 a	1.92 ± 0.16 a	7.99 ± 1.05 d	5.72 ± 0.55 d	10.37 ± 1.07 d
5	12	1.21 ± 0.08 c	12.16 ± 0.03 b	1.96 ± 0.16 a	9.28 ± 1.07 c	33.42 ± 2.97 c	29.67 ± 7.28 c
10	12	1.49 ± 0.12 b	11.89 ± 0.03 c	1.99 ± 0.16 a	10.78 ± 1.46 b	41.30 ± 6.99 b	48.96 ± 15.32 b
15	12	1.78 ± 0.11 a	11.71 ± 0.02 d	2.02 ± 0.21 a	12.54 ± 1.62 a	51.09 ± 8.74 a	67.97 ± 23.86 a
**Application**							
GG	16	1.42 ± 0.34 a	12.03 ± 0.27 a	1.95 ± 0.17 a	10.79 ± 1.98 a	31.85 ± 17.95 a	39.60 ± 26.46 ab
PG	16	1.28 ± 0.24 b	12.05 ± 0.28 a	2.00 ± 0.17 a	8.66 ± 1.72 b	33.08 ± 18.46 a	36.64 ± 25.11 b
PG + GG	16	1.43 ± 0.31 a	12.03 ± 0.26 a	1.96 ± 0.18 a	10.99 ± 1.99 a	33.72 ± 18.60 a	41.49 ± 27.61 a

Means with different letters within a column are significantly different (*p* < 0.05). n: number of samples analyzed according to (2 × 4 × 3) × 2 factorial design. CPT: Carrot powder type. CPR: Carrot powder ratio. GG: Guar gum. PG: Pregelatinization. PG + Gam: Pregelatinization and guar gum. Results are dry matter basis.

**Table 2:** Duncan’s multiple comparison test results of mineral content (mg/100 g) of gluten-free pasta samples.

Variance Source	n	Ca	Fe	K	Mg	P	Zn
**CPT**							
Orange	24	597.5 ± 237.2 b	2.13 ± 0.13 a	481.0 ± 99.8 b	94.77 ± 10.43 b	286.30 ± 4.02 b	2.26 ± 0.18 a
Black	24	614.1 ± 249.1 a	2.13 ± 0.12 a	557.1 ± 156.2 a	101.52 ± 14.89 a	292.27 ± 7.29 a	2.11 ± 0.17 b
**CPR (%)**							
0	12	290.94 ± 5.95 d	2.16 ± 0.12 a	358.26 ± 1.46 d	80.99 ± 0.50 d	283.79 ± 3.37 d	2.17 ± 0.16 a
5	12	491.25 ± 13.48 c	2.13 ± 0.10 a	451.42 ± 27.75 c	94.02 ± 4.12 c	287.90 ± 3.76 c	2.15 ± 0.16 a
10	12	716.26 ± 15.96 b	2.12 ± 0.14 a	577.70 ± 58.1 b	102.80 ± 4.70 b	291.53 ± 4.95 b	2.19 ± 0.19 a
15	12	924.75 ± 21.69 a	2.09 ± 0.12 a	688.80 ± 75.1 a	114.76 ± 6.10 a	293.93 ± 8.34 a	2.22 ± 0.26 a
**Application**							
GG	16	610.40 ± 246.8 a	2.16 ± 0.12 a	521.00 ± 138.9 a	97.80 ± 13.54 b	287.31 ± 5.93 b	2.21 ± 0.12 a
PG	16	595.30 ± 245.3 b	2.05 ± 0.10 a	514.20 ± 136.2 b	98.05 ± 13.07 ab	293.78 ± 5.97 a	2.10 ± 0.22 a
PG + GG	16	611.70 ± 245.7 a	2.17 ± 0.12 a	521.90 ± 139.4 a	98.58 ± 13.74 a	286.77 ± 5.60 b	2.24 ± 0.20 a

Means with different letters within a column are significantly different (*p* < 0.05). n: number of samples analyzed according to (2 × 4 × 3) × 2 factorial design. CPT: Carrot powder type. CPR: Carrot powder ratio GG: Guar gum. PG: Pregelatinization. PG + Gam: Pregelatinization and guar gum. Results are dry matter basis.

**Table 3:** β-carotene and total anthocyanin content of gluten-free pasta samples.

Application	CPR (%)	β-Karoten (mg/100 g) (Orange Carrot)	TA (mg CGE/100 g) (Black Carrot)
GG	0	nd	nd
	5	3.08 ± 0.03	20.87 ± 0.22
	10	3.33 ± 0.03	23.89 ± 0.17
	15	4.42 ± 0.06	26.08 ± 0.02
PG	0	nd	nd
	5	2.15 ± 0.04	15.46 ± 0.62
	10	2.67 ± 0.04	17.21 ± 0.29
	15	3.16 ± 0.06	18.53 ± 0.52
PG + GG	0	nd	nd
	5	2.27 ± 0.06	15.75 ± 0.27
	10	2.94 ± 0.06	17.27 ± 0.21
	15	3.24 ± 0.03	18.87 ± 0.17

Results are the average of two replications. CPR: Carrot powder ratio. TA: Total anthocyanin. GG: Guar gum. PG: Pregelatinization. PG + Gam: Pregelatinization and guar gum. nd: not detected. β-carotene analysis was performed on pasta samples containing orange carrot powder. Anthocyanin analysis was conducted on pasta samples containing black carrot powder. nd: not detected.

**Table 4:** Duncan’s multiple comparison test results of color values of gluten-free pasta samples.

Variance Source	n	L*	a*	b*	C*	Hueº
**CPT**						
Orange	24	81.79 ± 0.69 a	9.22 ± 5.10 a	27.14 ± 5.01 a	28.97 ± 5.73 a	77.42 ± 9.49 b
Black	24	62.46 ± 15.05 b	6.40 ± 3.93 b	3.13 ± 11.02 b	12.07 ± 6.15 b	277.60 ± 111.90 a
**CPR (%)**						
0	12	85.09 ± 1.30 a	0.70 ± 0.34 d	21.65 ± 1.25 a	21.67 ± 1.26 b	88.16 ± 0.77 c
5	12	71.99 ± 12.62 b	8.20 ± 3.08 c	12.01 ± 14.13 d	16.65 ± 11.72 d	206.10 ± 145.90 a
10	12	67.24 ± 14.55 c	10.64 ± 2.03 b	13.03 ± 17.36 c	20.72 ± 8.42 c	202.80 ± 142.10 b
15	12	64.18 ± 15.94 d	11.70 ± 1.10 a	13.84 ± 19.21 b	23.03 ± 12.23 a	203.10 ± 140.80 b
**Application**						
GG	16	77.00 ± 11.82 a	7.22 ± 4.68 c	13.21 ± 12.91 c	18.16 ± 8.84 c	174.60 ± 134.00 b
PG	16	69.75 ± 15.73 b	8.32 ± 4.80 a	16.52 ± 15.97 a	22.01 ± 11.24 a	176.60 ± 134.60 a
PG + GG	16	69.62 ± 15.45 b	7.88 ± 4.93 b	15.67 ± 16.06 b	21.38 ± 11.09 b	173.90 ± 130.10 b

Means with different letters within a column are significantly different (*p* < 0.05). n: number of samples analyzed according to (2 × 4 × 3) × 2 factorial design. CPT: Carrot powder type. CPR: Carrot powder ratio. GG: Guar gum. PG: Pregelatinization. PG + Gam: Pregelatinization and guar gum.

**Table 5:** Duncan’s multiple comparison test results of cooking properties and firmness values of gluten-free pasta samples.

Variance Source	n	Wight Increase (%)	Volume Increase (%)	Cooking Loss (%)	Firmness (g)
**CPT**					
Orange	24	121.26 ± 18.17 b	121.17 ± 21.89 b	5.88 ± 1.69 b	65.80 ± 8.35 b
Black	24	136.94 ± 17.72 a	134.83 ± 25.45 a	6.40 ± 2.09 a	105.13 ± 33.73 a
**CPR (%)**					
0	12	120.08 ± 11.92 c	109.17 ± 22.66 c	5.26 ± 1.28 b	56.84 ± 0.49 d
5	12	126.30 ± 18.08 b	127.17 ± 23.09 b	6.06 ± 1.93 ab	97.90 ± 36.5 a
10	12	133.94 ± 21.32 a	136.00 ± 19.58 a	6.42 ± 1.89 a	96.22 ± 31.56 b
15	12	136.08 ± 22.71 a	139.67 ± 22.87 a	6.79 ± 2.26 a	90.86 ± 25.70 c
**Application**					
GG	16	149.52 ± 12.39 a	153.00 ± 14.17 a	8.37 ± 1.30 a	76.90 ± 23.04 c
PG	16	124.54 ± 10.94 b	124.00 ± 15.71 b	5.38 ± 0.94 b	81.83 ± 27.96 b
PG + GG	16	113.24 ± 13.28 c	107.00 ± 16.59 c	4.65 ± 0.65 b	97.65 ± 39.06 a

Means with different letters within a column are significantly different (*p* < 0.05). n: number of samples analyzed according to (2 × 4 × 3) × 2 factorial design. CPT: Carrot powder type. CPR: Carrot powder ratio. GG: Guar gum. PG: Pregelatinization. PG + Gam: Pregelatinization and guar gum.

## Data Availability

The original contributions presented in this study are included in the article/Supplementary Materials. Further inquiries can be directed to the corresponding author.

## Supplementary Materials

The following supporting information can be downloaded at: https://www.mdpi.com/article/10.3390/foods13244101/s1, Table S1. Experimental designe and ingredients of gluten-free pasta samples; Table S2. Chemical and bioactive component analysis results of raw materials used in gluten-free pasta production; Table S3. Mineral matter (mg/100g) results of raw materials used in gluten-free pasta production; Table S4. Color values of raw materials used in gluten-free pasta production; Table S5. Chemical and bioactive component analysis results of gluten-free pasta samples; Table S6. Mineral (mg/100g) analysis results of gluten-free pasta samples; Table S7. Color values of gluten-free pasta samples; Table S8. Cooking properties and firmness values of gluten-free pasta samples; Figure S1. Gluten-free pasta samples prepared with orange carrot powder (OCP) and PG+GG application; Figure S2. Gluten-free pasta samples prepared with black carrot powder (BCP) and PG+GG application.
