# Supplementary material for: Improvement of Bioactive Components and Technological Quality of Gluten-Free Pasta with Utilization of Different Carrot Powders, Guar Gum and Pregelatinization Application
Source: Foods. 2024 Dec 18;13(24):4101. doi: 10.3390/foods13244101 (PMC11675153; doi:10.3390/foods13244101)
Supplement: Supplementary file 1 [file foods-13-04101-s001.zip › foods-3359180-supplementary.pdf]

Supplementary Materials

Table S1. Experimental designe and ingredients of gluten-free pasta samples

CPT	Application	CPR (%)	Corn flour	Rice flour	Chickpea flour	GG	Orange carrot flour	Black carrot flour
Orange	GG	**0**	38.5	38.5	20	3	0	-
		**5**	36	36	20	3	5	-
		**10**	33.5	33.5	20	3	10	-
		**15**	31	31	20	3	15	-
	PG	**0**	40	40	20	-	0	-
		**5**	37.5	37.5	20	-	5	-
		**10**	35	35	20	-	10	-
		**15**	32.5	32.5	20	-	15	-
	PG+GG	**0**	38.5	38.5	20	3	0	-
		**5**	36	36	20	3	5	-
		**10**	33.5	33.5	20	3	10	-
		**15**	31	31	20	3	15	-
Black	GG	**0**	38.5	38.5	20	3	-	0
		**5**	36	36	20	3	-	5
		**10**	33.5	33.5	20	3	-	10
		**15**	31	31	20	3	-	15
	PG	**0**	40	40	20	-	-	0
		**5**	37.5	37.5	20	-	-	5
		**10**	35	35	20	-	-	10
		**15**	32.5	32.5	20	-	-	15
	PG+GG	**0**	38.5	38.5	20	3	-	0
		**5**	36	36	20	3	-	5
		**10**	33.5	33.5	20	3	-	10
		**15**	31	31	20	3	-	15

CPT: Carrot powder type. CPR: Carrot powder ratio GG: Guar gum. PG: Pregelatinization. PG+Gam: Pregelatinization and guar gum

Table S2. Chemical and bioactive component analysis results of raw materials used in gluten-free pasta production

Raw material	Ash (%)	Protein (%)	Fat (%)	Antioxidant activity (%)	TPC (mgGAE/100g)	TDF (%)
**Corn flour**	0.65±0.06c	9.00±1.10b	1.71±0.25b	14.08±1.60c	27.03±13.01c	4.49±0.47d
**Rice flour**	0.42±0.04c	10.79±1.63b	0.59±0.04b	2.66±0.58d	10.96±3.22c	2.78±0.20d
**Chickpea flour**	2.88±0.27b	24.29±2.46a	5.38±0.75a	24.08±2.56c	33.35±1.58c	17.01±0.55c
**Orange carrot powder**	5.71±0.07a	7.93±1.95b	2.16±0.25b	75.16±5.40b	263.50±5.50b	29.27±2.49b
**Black carrot powder**	5.88±0.92a	8.49±0.52b	2.55±0.76b	87.52±1.19a	596.10±16.7a	42.92±2.91a

Means with the different letter within a column are significantly different ($p < 0.05$). TPC: Total phenolic content. TDF: Total dietary fiber. Results are dry matter basis.

Table S3. Mineral matter (mg/100g) results of raw materials used in gluten-free pasta production

Raw material	Ca	Fe	K	Mg	P	Zn
**Corn flour**	12.99±0.62d	0.47±0.013d	188.93±3.35d	62.52±0.89d	184.44±2.69d	0.98±0.03d
**Rice flour**	15.37±1.59d	0.24±0.02d	117.92±7.59d	27.44±0.45e	196.87±3.27d	1.74±0.04c
**Chickpea flour**	81.80±1.65c	8.94±0.26a	1202.00±123c	176.71±3.47c	528.40±23.90a	4.68±0.31a
**Orange carrot powder**	403.48±3.18b	1.30±0.06c	1630.90±58.8b	224.23±5.47b	293.19±4.16c	2.92±0.14b
**Black carrot powder**	418.13±3.20a	1.89±0.03b	2296.90±101a	296.00±14.90a	361.48±8.79b	1.42±0.23cd

Means with the different letter within a column are significantly different ($p < 0.05$). Results are dry matter basis.

Table S4. Color values of raw materials used in gluten-free pasta production

Raw material	L*	a*	b*	C*	Hue°
**Corn flour**	91.75±3.59a	-0.50±0.07c	23.68±1.32b	23.68±1.32b	91.21±0.11b
**Rice flour**	94.50±3.18a	0.84±0.02c	5.15±0.42c	5.22±0.41d	80.68±0.52c
**Chickpea flour**	89.44±1.09ab	0.30±0.05c	21.98±2.47b	21.98±2.48b	89.23±0.05b
**Orange carrot powder**	70.81±2.07b	19.20±1.34a	35.75±1.94a	40.58±2.35a	61.78±0.37d
**Black carrot powder**	39.28±2.59c	12.19±0.10b	-4.02±0.15d	12.84±0.05c	341.73±0.77a

Means with the different letter within a column are significantly different ($p < 0.05$).

Table S5. Chemical and bioactive component analysis results of gluten-free pasta samples

CPT	Application	CPR (%)	Ash (%)	Protein (%)	Fat (%)	TDF (%)	Antioxidant activity (%)	TPC (mgGAE/100g)
Orange	GG	0	1.04±0.01	12.38±0.03	1.91±0.23	8.49±0.40	5.27±0.34	10.06±1.99
		5	1.23±0.01	12.15±0.02	1.93±0.16	9.70±0.40	30.53±2.23	24.81±4.29
		10	1.56±0.04	11.86±0.06	1.96±0.23	10.23±0.48	33.83±3.64	36.43±3.93
		15	1.87±0.01	11.71±0.05	1.99±0.34	12.75±0.71	42.06±4.93	49.67±2.42
	PG	0	1.02±0.00	12.42±0.01	1.95±0.24	6.71±0.35	5.84±0.17	9.58±0.65
		5	1.13±0.02	12.16±0.06	1.98±0.31	7.88±0.90	31.52±2.97	19.84±2.43
		10	1.32±0.02	11.89±0.01	2.00±0.27	8.85±0.16	35.88±3.15	31.02±2.64
		15	1.63±0.02	11.71±0.02	2.03±0.28	10.03±0.50	43.33±2.94	43.15±3.96
	PG+GG	0	1.05±0.01	12.37±0.02	1.90±0.27	8.55±0.37	6.17±0.556	10.86±1.77
		5	1.28±0.04	12.13±0.04	1.92±0.18	9.96±0.16	32.59±3.31	27.86±0.18
		10	1.54±0.06	11.87±0.02	1.95±0.13	11.01±0.28	35.31±2.33	37.07±4.16
		15	1.85±0.05	11.70±0.01	1.98±0.37	12.14±0.18	44.08±3.57	44.07±4.29
Black	GG	0	1.04±0.01	12.38±0.05	1.89±0.21	8.78±0.20	5.25±0.34	10.81±0.59
		5	1.18±0.07	12.17±0.03	1.95±0.18	9.97±0.40	34.07±1.16	32.32±5.50
		10	1.60±0.00	11.88±0.01	1.98±0.10	12.32±0.43	46.58±2.55	62.23±6.41
		15	1.87±0.02	11.70±0.03	2.02±0.28	14.08±0.55	57.20±2.52	90.46±4.68
	PG	0	1.02±0.00	12.40±0.04	1.93±0.18	6.52±0.29	5.75±0.89	9.84±0.55
		5	1.15±0.03	12.18±0.01	1.99±0.18	7.98±0.40	35.23±2.78	34.47±5.36
		10	1.36±0.04	11.90±0.02	2.04±0.17	9.65±0.44	47.30±3.38	60.64±4.50
		15	1.64±0.03	11.71±0.01	2.07±0.14	11.68±0.24	59.79±3.61	84.58±2.52
	PG+GG	0	1.04±0.00	12.36±0.02	1.92±0.11	8.86±0.34	6.05±0.51	11.06±0.81
		5	1.31±0.03	12.16±0.04	1.96±0.20	10.21±0.34	36.58±3.30	38.70±5.46
		10	1.56±0.00	11.91±0.03	1.99±0.30	12.61±0.65	48.89±2.49	66.40±2.59
		15	1.84±0.00	11.74±0.02	2.03±0.21	14.59±0.48	60.05±2.73	95.89±5.43

Results are the average of two replication. CPT: Carrot powder type. CPR: Carrot powder rati.o TDF : Total dietary fiber TPC: Total phenolic content. GG: Guar gum. PG: Pregelatinization. PG+Gam: Pregelatinization and guar gum

Table S6. Mineral (mg/100g) analysis results of gluten-free pasta samples

CPT	Application	CPR (%)	Ca	Fe	K	Mg	P	Zn
Orange	GG	0	295.00±2.83	2.18±0.08	359.46±0.20	81.53±0.09	281.40±2.12	2.15±0.18
		5	488.00±8.49	2.13±0.08	424.48±0.28	90.07±0.68	283.50±1.34	2.20±0.11
		10	718.90±23.8	2.20±0.21	523.61±0.89	98.40±0.10	285.50±1.34	2.29±0.18
		15	909.99±8.34	2.09±0.16	617.72±1.75	108.91±0.52	287.60±0.31	2.36±0.16
	PG	0	281.01±1.86	2.20±0.23	358.29±0.24	81.50±0.39	288.30±0.31	2.09±0.08
		5	471.01±5.68	2.09±0.11	420.40±1.30	90.20±0.68	289.80±1.37	2.00±0.04
		10	688.31±4.22	2.04±0.11	516.99±0.04	97.80±0.10	291.60±2.20	2.25±0.17
		15	895.00±7.07	2.01±0.13	614.90±0.53	108.50±0.82	293.40±0.31	2.39±0.44
	PG+GG	0	296.62±2.47	2.21±0.16	359.52±0.57	80.72±0.09	282.23±1.34	2.23±0.11
		5	487.96±3.30	2.18±0.17	431.70±0.28	91.09±0.69	284.80±1.35	2.30±0.11
		10	726.50±7.18	2.13±0.16	526.70±2.63	99.00±0.47	286.30±0.30	2.37±0.16
		15	911.50±5.28	2.09±0.17	618.58±0.14	109.46±0.83	280.90±1.33	2.45±0.14
Black	GG	0	292.00±4.82	2.19±0.11	358.30±0.31	80.64±0.38	289.90±1.33	2.16±0.14
		5	507.51±3.32	2.18±0.14	486.50±2.06	94.43±0.10	287.20±1.36	2.22±0.16
		10	719.75±1.96	2.17±0.25	632.97±0.58	107.80±0.81	293.56±0.31	2.18±0.03
		15	952.01±1.98	2.16±0.14	765.28±0.86	120.60±0.57	298.50±1.42	2.14±0.06
	PG	0	287.00±1.44	2.00±0.07	356.09±0.10	81.02±0.61	287.86±1.37	2.17±0.30
		5	487.01±1.95	2.04±0.01	470.20±0.96	99.71±0.11	294.10±2.22	1.99±0.00
		10	715.00±1.44	2.03±0.01	623.68±0.43	105.60±0.50	299.60±1.42	1.96±1.16
		15	938.00±1.77	2.02±0.03	752.88±1.25	120.10±0.91	305.60±0.32	1.92±0.10
	PG+GG	0	293.99±0.61	2.20±0.06	357.92±2.70	80.53±0.38	282.02±1.34	2.23±0.31
		5	506.00±2.84	2.19±2.11	475.27±2.25	98.62±0.74	288.00±0.31	2.17±0.28
		10	729.05±4.30	2.18±0.16	642.31±0.68	108.20±0.82	292.30±1.39	2.11±0.24
		15	942.01±3.45	2.17±0.17	763.50±5.76	121.00±0.13	297.59±0.32	2.06±0.21

Results are the average of two replication. CPT: Carrot powder type. CPR: Carrot powder ratio GG: Guar gum. PG: Pregelatinization. PG+Gam: Pregelatinization and guar gum

Table S7. Color values of gluten-free pasta samples

CPT	Application	CPR (%)	L*	a*	b*	C*	Hue°
Orange	GG	0	86.45±0.65	0.45±0.00	20.16±0.15	20.16±0.15	88.72±0.00
		5	85.65±0.10	9.84±0.05	22.25±0.11	24.33±0.11	66.14±0.00
		10	84.42±0.09	12.11±0.01	24.46±0.03	27.29±0.03	63.65±0.00
		15	83.09±0.63	12.47±0.01	26.62±0.20	29.40±0.19	64.90±0.14
	PG	0	84.20±0.09	1.12±0.00	23.03±0.11	23.06±0.11	87.22±0.00
		5	81.48±0.09	11.87±0.09	27.36±0.03	29.83±0.06	66.56±0.14
		10	78.69±0.59	12.91±0.06	31.99±0.03	34.50±0.05	68.03±0.07
		15	77.51±0.08	12.66±0.06	34.98±0.17	37.21±0.18	70.10±0.00
	PG+GG	0	84.15±0.63	0.50±0.00	21.73±0.10	21.73±0.10	88.69±0.00
		5	81.50±0.39	11.45±0.09	26.67±0.20	29.02±0.22	66.77±0.00
		10	78.31±0.08	12.54±0.09	31.93±0.24	34.30±0.26	68.55±0.00
		15	76.02±0.36	12.73±0.10	34.49±0.16	36.76±0.19	69.74±0.05
Black	GG	0	87.02±0.66	0.47±0.00	20.23±0.10	20.24±0.10	88.67±0.00
		5	71.23±0.54	4.60±0.02	-0.99±0.00	4.71±0.02	347.91±0.03
		10	61.64±0.46	7.95±0.04	-2.85±0.01	8.45±0.04	340.22±0.00
		15	56.49±0.43	9.86±0.01	-4.23±0.00	10.73±0.01	336.80±0.00
	PG	0	84.45±0.64	1.21±0.00	23.21±0.11	23.24±0.11	87.02±0.00
		5	55.96±0.26	5.81±0.03	-0.61±0.00	5.84±0.03	354.04±0.02
		10	49.30±0.05	9.46±0.01	-3.36±0.02	10.04±0.02	340.46±0.12
		15	46.42±0.35	11.56±0.01	-4.43±0.00	12.38±0.01	339.04±0.00
	PG+GG	0	84.28±0.40	0.50±0.00	21.57±0.00	21.57±0.10	88.67±0.00
		5	56.13±0.06	5.58±0.03	-2.61±0.00	6.16±0.03	334.91±0.08
		10	51.06±0.38	8.89±0.01	-3.96±0.02	9.73±0.02	336.00±0.08
		15	45.54±0.22	10.87±0.01	-4.42±0.00	11.74±0.01	337.87±0.00

Results are the average of two replication. CPT: Carrot powder type. CPR: Carrot powder ratio. GG: Guar gum. PG: Pregelatinization. PG+Gam: Pregelatinization and guar gum

Table S8. Cooking properties and firmness values of gluten-free pasta samples

CPT	Application	CPR	Wight increase (%)	Volume increase (%)	Cooking loss (%)	Firmness (g)
Orange	GG	0	134.25±1.36	138.00±2.29	6.72±1.07	56.84±0.27
		5	141.60±3.75	143.00±2.53	8.15±0.42	61.60±0.06
		10	149.23±1.32	148.00±4.86	8.34±0.34	62.81±0.47
		15	150.00±2.67	150.00±3.03	8.65±0.34	65.99±0.07
	PG	0	115.80±1.29	99.00±4.54	4.77±1.08	56.35±0.42
		5	113.26±1.43	125.00±4.70	4.62±0.93	62.18±0.07
		10	118.65±3.30	128.00±3.03	5.53±0.70	65.89±0.50
		15	122.36±3.76	130.00±3.80	5.35±0.69	69.26±0.07
	PG+GG	0	108.50±3.08	88.00±6.11	4.31±0.95	57.32±0.43
		5	98.50±3.18	90.00±5.70	4.56±0.83	68.05±0.32
		10	101.85±3.14	105.00±3.95	4.69±0.46	80.83±0.09
		15	101.15±1.65	110.00±4.54	4.81±0.76	82.46±0.62
Black	GG	0	136.55±4.70	140.00±2.04	6.71±0.25	56.84±0.43
		5	150.25±4.43	160.00±6.10	8.87±1.02	118.59±0.40
		10	164.05±3.99	165.00±3.48	9.20±0.90	104.49±0.78
		15	170.23±3.01	180.00±3.45	10.34±0.93	88.06±0.09
	PG	0	115.90±2.53	100.00±1.73	4.76±1.05	56.35±0.27
		5	128.90±4.60	130.00±3.96	5.26±0.94	130.99±0.44
		10	140.50±2.91	140.00±3.04	5.90±0.94	113.95±0.12
		15	140.95±2.98	140.00±2.04	6.84±0.69	99.67±0.47
	PG+GG	0	109.50±3.56	90.00±2.83	4.32±0.75	57.32±0.27
		5	125.30±3.32	115.00±2.83	4.92±0.98	146.17±0.78
		10	129.35±4.38	130.00±4.99	4.85±0.85	149.33±0.71
		15	131.80±3.28	128.00±2.80	4.74±1.01	139.75±1.05

Results are the average of two replication. CPT: Carrot powder type. CPR: Carrot powder ratio. GG: Guar gum. PG: Pregelatinization. PG+Gam: Pregelatinization and guar gum

%0 OCP

%5 OCP

%10 OCP

%15 OCP

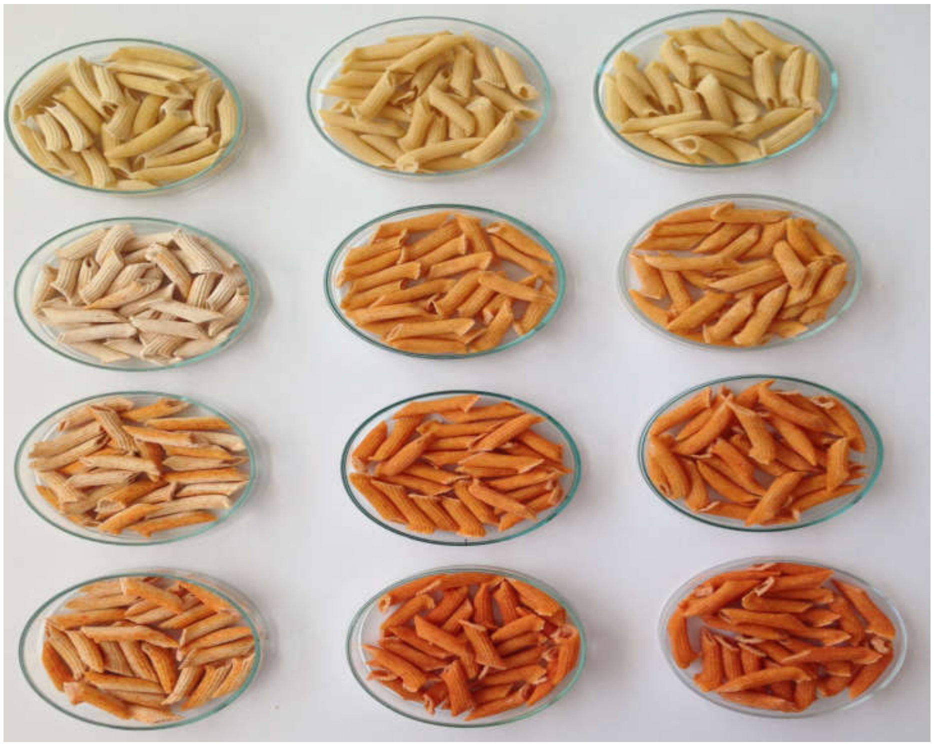

Figure S1. Gluten-free pasta samples prepared with orange carrot powder (OCP) and PG+GG application

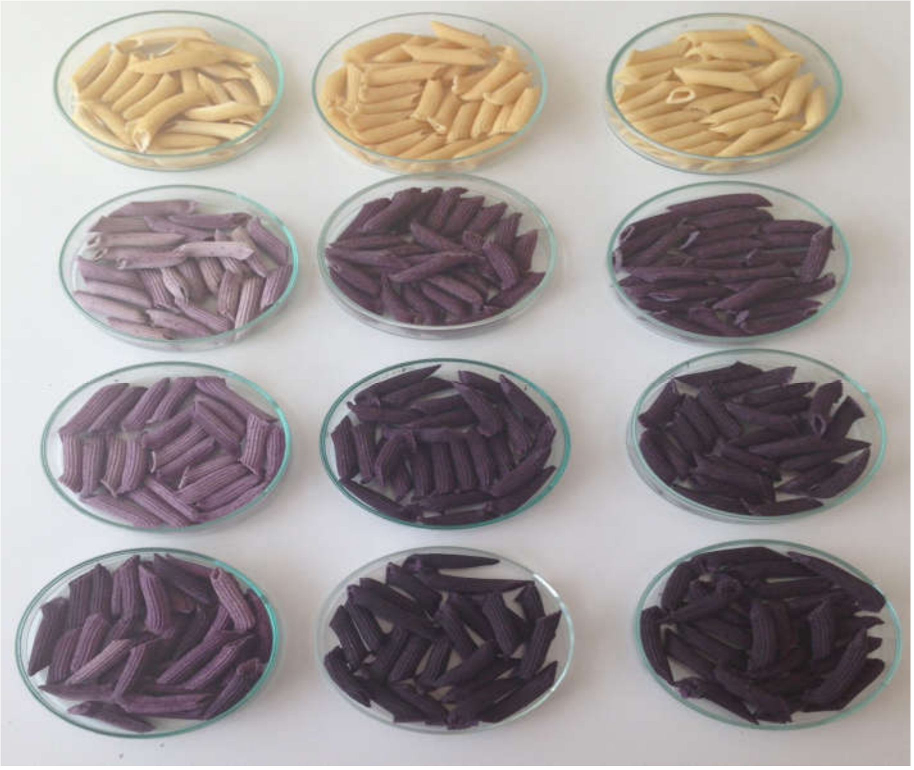

Figure S2. Gluten-free pasta samples prepared with black carrot powder (BCP) and PG+GG application
